# UPLC-MS-Based Serum Metabolomics Reveals Potential Biomarkers of Ang II-Induced Hypertension in Mice

**DOI:** 10.3389/fcvm.2021.683859

**Published:** 2021-05-05

**Authors:** Shaying Yang, Zhiwei Wang, Mengting Guo, Mengfan Du, Xin Wen, Li Geng, Fan Yu, Liangliang Liu, Yanting Li, Lei Feng, Tingting Zhou

**Affiliations:** Laboratory of Cardiovascular Research, School of Medicine, Jiangnan University, Wuxi, China

**Keywords:** hypertension, LC-MS, angiotensin II, metabolomics, serum metabolites, biomarkers, mice

## Abstract

Hypertension is caused by polygenic inheritance and the interaction of various environmental factors. Abnormal function of the renin-angiotensin-aldosterone system (RAAS) is closely associated with changes in blood pressure. As an essential factor in the RAAS, angiotensin II (Ang II) contributes to vasoconstriction and inflammatory responses. However, the effects of overproduction of Ang II on the whole body-metabolism have been unclear. In this study, we established a hypertensive mouse model by micro-osmotic pump perfusion of Ang II, and the maximum systolic blood pressure reached 140 mmHg after 2 weeks. By ultra-performance liquid chromatography-quadrupole time-of-flight mass spectrometry, the metabolites in the serum of hypertensive model and control mice were analyzed. Partial least squares discriminant analysis (PLS-DA) in both positive and negative ionization modes showed clear separation of the two groups. Perfusion of Ang II induced perturbations of multiple metabolic pathways in mice, such as steroid hormone biosynthesis and galactose metabolism. Tandem mass spectrometry revealed 40 metabolite markers with potential diagnostic value for hypertension. Our data indicate that non-targeted metabolomics can reveal biochemical pathways associated with Ang II-induced hypertension. Although researches about the clinical use of these metabolites as potential biomarkers in hypertension is still needed, the current study improves the understanding of systemic metabolic response to sustained release of Ang II in hypertensive mice, providing a new panel of biomarkers that may be used to predict blood pressure fluctuations in the early stages of hypertension.

## Introduction

Hypertension is a common cardiovascular disease and the leading risk factor for both cardiovascular and cerebrovascular events. It can cause functional or organic lesions of the heart, brain, blood vessels, kidneys, and other organs, contributing to a significant cause of disability and death (1). The incidence and development of hypertension are affected by both genetic and environmental factors (2). In recent studies, 60% of the main factors leading to hypertension have been associated with metabolic abnormalities, while 80% of hypertensive patients have various forms of metabolic disorder (3).

Ang II increases systemic blood pressure and glomerular capillary pressure. It is directly involved in renal arteriosclerosis and causes kidney damage (4). It also increases the pressure in the glomeruli and contracts mesangial cells, leading to an increase in the selective permeability to urine proteins. Clinical and experimental studies have shown that it regulates the processes of inflammation and fibrosis contributing to kidney pathogenesis through activating growth factors associated with fibrosis (5). The clinical manifestations of hypertension and kidney damage are persistent hypertension accompanied by persistent trace or mild-to-moderate proteinuria, and impaired renal function (i.e., increased creatinine and urea nitrogen) (6). Ang II-induced hypertension leads to the hypertrophy of smooth muscle cells and to increases in the expression of their specific markers, eventually leading to thickening of the arterial media and increasing vascular resistance (7).

A study revealed that Ang II induction increases thromboxane production in mice (8), and another indicated that prolonged Ang II-induced hypertension and massive blood-brain barrier leakage, microglia activation, myelin loss, and memory dysfunction are associated with stroke compared with control mice (9).

High-throughput full-spectrum analysis of metabolites provides an opportunity to assess disease severity, restored metabolic pathways, and homeostasis (10, 11). Identifying the disturbed biochemical pathways helps to understand changes in body components during the development of hypertension. Therefore, we aimed to find the endogenous molecular metabolites regulating the blood vessels of mice during the induction by Ang II by analyzing the metabolic spectrum that can be used as a marker for early blood pressure fluctuations.

Metabolomics based on liquid chromatography-mass spectrometry (LC-MS) is an effective method for the metabolic profiling of biological systems (12). LC-MS analysis has higher sensitivity and a more comprehensive polarity range than NMR spectroscopy (13, 14). In the current study, the UPLC-Q-TOF/MS (ultra-performance liquid chromatography-quadrupole time-of-flight mass spectrometry) platform was used to analyze serum samples from control and Ang II-induced hypertensive mice to explore the differential metabolites of hypertension induced by slow-release Ang II. We identified >40 different metabolites involved in >20 metabolic pathways in the Ang II mice.

## Methods and Materials

### Animals and Sample Collection

All animal experiments were performed in accordance with the laboratory animal guidelines and with the approval of the Animal Experimentation Ethics Committee, Jiangnan University (License No: JN. No 20190930c1000120[232]). The male C57BL/6J mice used in the experiments were provided by the Institute of Model Animal Research of Nanjing University, and were reared in a specific pathogen-free environment. The average body weight was ~23 g. The mice were divided into two groups (Ang II and control groups) of 10 mice each. Mice were infused subcutaneously with Ang-II (Sigma, 600 ng/kg/min), or vehicle (0.9% saline) for 19 days using osmotic pumps (Alzet) (15). Venous blood was collected from the retro-orbital venous plexus using a blood collection capillary.

### Measurement of Blood Pressure

We used a non-invasive blood pressure meter (IITC Life Science) to measure the blood pressure of the mice.

### Sample Preparation

Two hundred microliters of each sample was placed in a new Eppendorf tube, to which was added 800 μL of methanol/water (v/v = 8:1) pre-cooled at −20°C for >0.5 h; then iron beads were added and the samples were lysed at 60 Hz for 5 min. After each sample is mixed in equal volume, it is used as a quality control (QC) sample.

### Metabolomic Analysis Based on LC-MS/MS

An ACQUITY UPLC BEH C18 column (100 × 2.1 mm, 1.7 μm, Waters Corp., UK) was used for chromatographic separation. The column temperature was 50°C and the flow rate was 0.4 ml/min, where mobile phase A was water and 0.1% formic acid, and mobile phase B was methanol and 0.1% formic acid. The sample was eluted with the following gradient: 0–2 min, 100% A; 2–11 min, 0–100% B; 11–13 min, 100% B; 13–15 min 0–100% A. For the small molecules eluted from the column, the high-resolution tandem mass spectrometry using Xevo G2-XS QTOF (Waters, UK) was used to collect positive and negative ions. In electrospray ionization positive ion mode (ESI+), the capillary voltage was 3 kV and the cone voltage 40 V; in ESI negative ion mode (ESI–), the capillary and cone voltages were 1 kV and 40 V. The mass spectrometry data were acquired in Centroid MSE mode. The TOF mass range was from 50 to 1,200 Da and the scan time was 0.2 s. For the MS/MS detection, all precursors were fragmented using 20–40 eV, and the scan time was 0.2 s. During the acquisition, the LE signal was acquired every 3 s to calibrate the mass accuracy. Meanwhile, the quality control (QC) samples were collected every 10 samples to evaluate the stability of the instrument during the sample collection process.

### Data Processing, Mass Spectrometric Identification, and Statistical Analysis

Peak extraction was mainly achieved using the software Progenesis QI (version 2.2), including peak alignment, peak extraction, normalization, deconvolution, and compound identification. See the previous report for details (16). The results show the mean ± SEM. Comparisons among groups were made using ANOVA or unpaired Student's *t*-test, with *P* < 0.05 as the threshold for a significant difference.

### Metabolite Annotation and Pathway Analysis

Metabolites were identified by matching the exact molecular mass data (m/z) of the samples against METLIN (http://metlin.scripps.edu/) and the Human Metabolome Database (http://www.hmdb.ca/) with 10-ppm accuracy (17, 18). We quantitatively mapped the different metabolites to the reference paths in the online Kyoto Encyclopedia of Genes and Genomes database (https://www.kegg.jp/kegg/pathway.html). Statistically significant enrichment pathways were evaluated by the hypergeometric test adjusted by the false discovery rate (*P* < 0.05).

## Results

### Mouse Model of Ang II-Induced Hypertension

**Figure 1:**
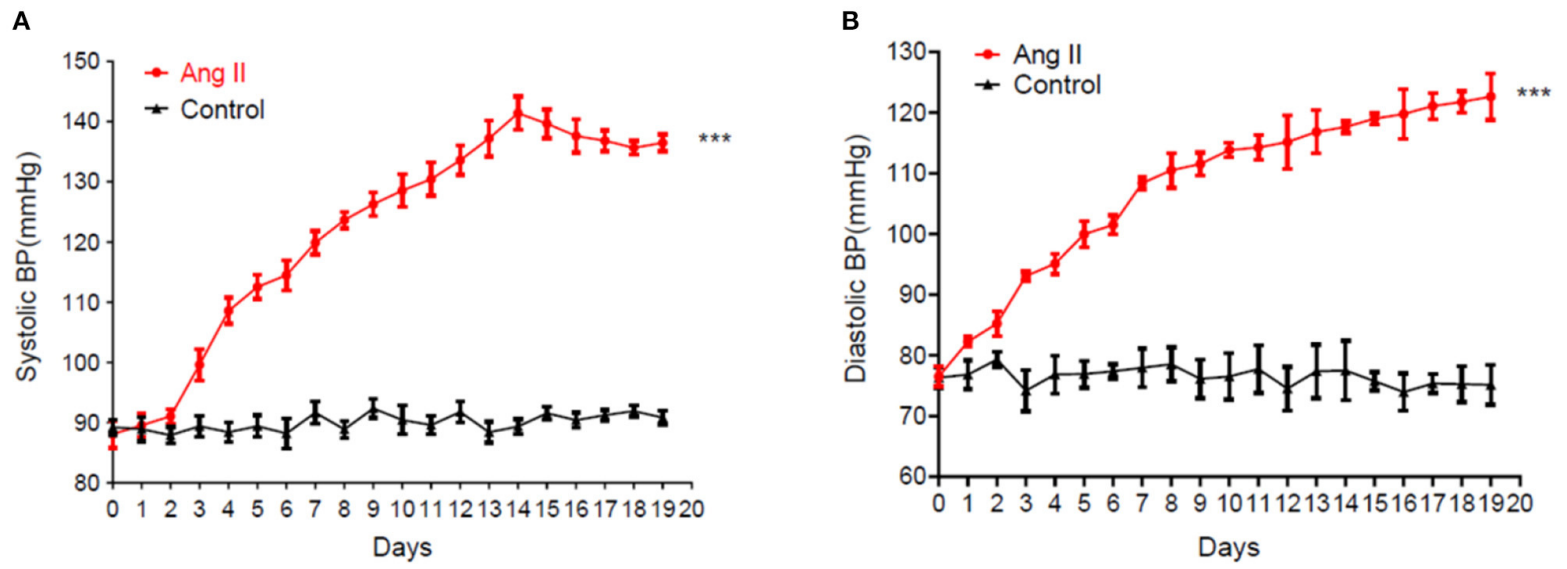
Sustained rise of systolic blood pressure **(A)** and diastolic blood pressure **(B)** in mice induced by the continuous release of Ang II (data are the mean ± SEM, *n* = 10, ***P < 0.001 vs. Control, two-way ANOVA).

Blood pressure recorded in the Ang II model significantly and continuously rose from day 5 (Figure 1). On day 17, the systolic blood pressure in the Ang II group was 140 mmHg while the controls remained at ~89 mmHg. Therefore, this model of hypertension induced by slow-release Ang II was successful.

### Metabolites Differ Between Control and Ang II-Induced Hypertensive Mice

**Figure 2:**
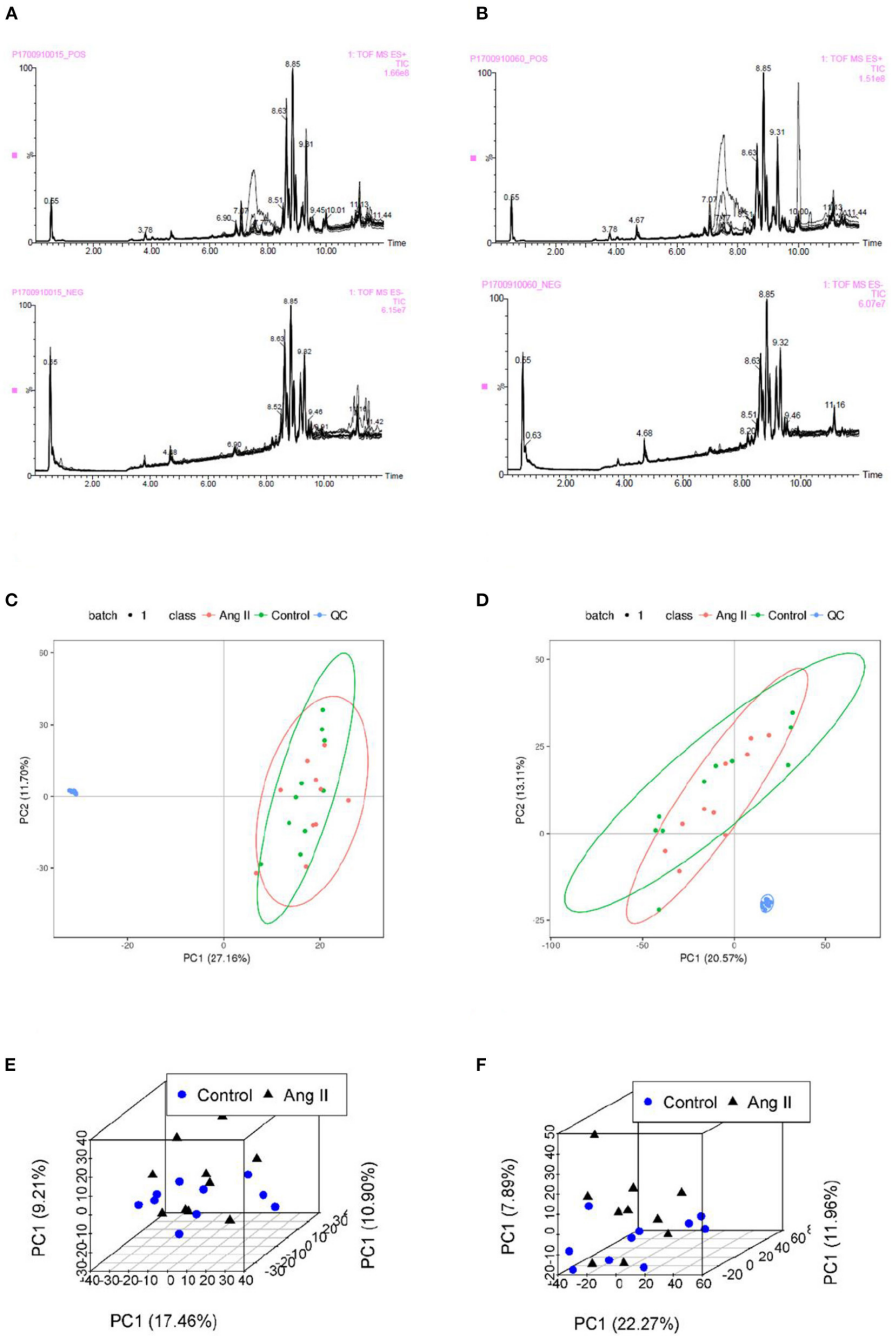
**(A,B)** Total ion chromatograms of QC samples in Control **(A)** and Ang II groups **(B)** in ESI+ and ESI– mode. **(C,D)** Plots of PCA scores for serum samples from test mice and QC samples showing metabolites obtained in ESI+ mode **(C)** and ESI– mode **(D)**. **(E,F)** Scatter plots of PCA scores of metabolites from the LC-MS/MS fingerprints in ESI+ mode **(E)** and ESI– mode **(F)**.

**Figure 3:**
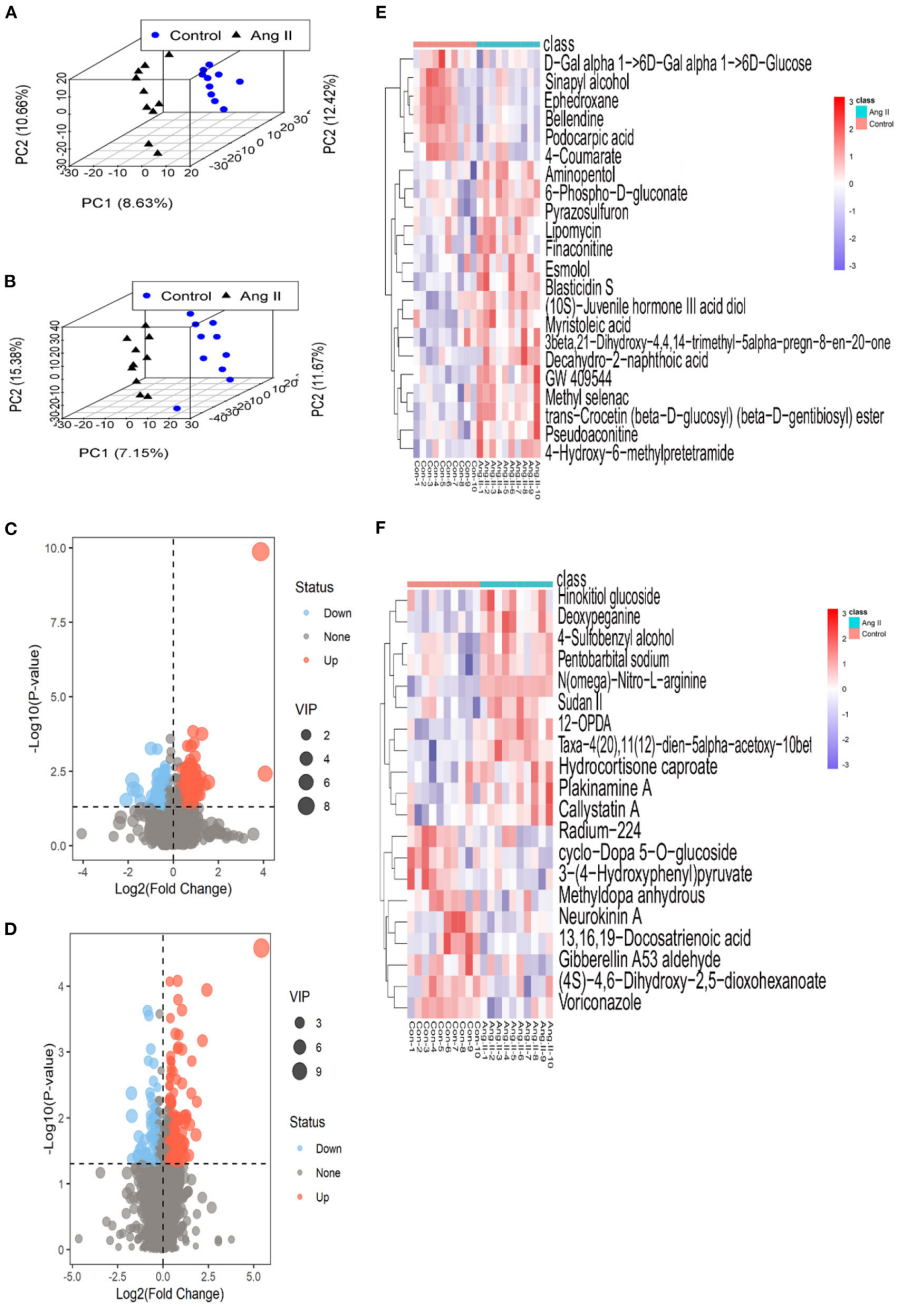
**(A,B)** 3-D plots of scores from partial least-squares discriminant analysis based on the metabolic profiling data from the plasma of Ang II-induced hypertensive mice and healthy (control) mice in ESI+ **(A)** and ESI– mode **(B)** (black triangles, Ang II-induced hypertensive mice; blue circles, control mice). **(C,D)** Volcano plots based on *P*-values and fold-changes of single-dimensional tests in ESI+ mode **(C)** and ESI– mode **(D)**. **(E,F)** Heatmaps of the differential metabolites for Ang II vs. control in ESI+ mode **(E)** and ESI– mode **(F)**.

None of the QC sample chromatograms showed significant retention time drift. We measured 5,904 ions in ESI+ mode and 6,937 in ESI– mode (Figures 2A,B). After elimination and filling in, 4,557 and 5,773 ions were finally obtained. The QC samples were tightly clustered, and were significantly separate from the test samples, indicating that the LC-MS/MS analysis platform had high stability and reproducibility (16) (Figures 2C,D). We further used three-dimensional principal component analysis (PCA) scatter plots to evaluate changes in the metabolite profile of mice during the development of Ang II-induced hypertension. The ordinary “unsupervised” analysis was unable to distinguish between the Ang II and control groups (Figures 2E,F). However, the use of partial least-squares discriminant analysis built an excellent regression model. The three-dimensional scatter diagram showed that the Ang II and control groups were significantly separated (Figures 3A,B), showing different metabolic phenotypes. This indicated that the sustained release of Ang II leads to metabolic disorders in mice. We selected the top 20 serum metabolites that met the variable importance in the projection threshold (VIP > 1) and Student's *t*-test (*P* < 0.05) criteria in the ESI+ and ESI– modes. Volcano maps based on *P*-values and one-dimensional test multiple changes (Figures 3C,D) and heat maps based on differences in metabolite abundance also showed clear separation of the Ang II and control groups (Figures 3E,F).

In ESI+ mode, contents of 4-Hydroxy-6-methylpretetramide, 6-Phospho-D-gluconate and Aminopentol considerably increased, and Ephedroxane and Bellendine obviously decreased in Ang II-induced hypertensive mice (Table 1). Likewise, variance in the production of N(omega)-Nitro-L-arginine, Deoxypeganine and Hinokitiol glucoside was also observed between Ang II and control groups in ESI- mode (Table 2).

**Table 1:** Top 20 differential serum metabolites between Ang II-induced hypertension mice and control mice in ESI+ mode.

**Compound**	***P*-value**	**Regulation**	**Fold change**	**Retention time/min**
(10S)-Juvenile hormone III acid diol	0.009	Up	2.001	5.587
3beta,21-Dihydroxy-4,4,14-trimethyl-5alpha-pregn-8-en-20-one	0.020	Up	2.249	7.477
4-Coumarate	0.043	Down	0.527	4.582
4-Hydroxy-6-methylpretetramide	0.004	Up	16.918	6.664
6-Phospho-D-gluconate	0.007	Up	2.917	4.511
Aminopentol	0.009	Up	2.648	6.807
Bellendine	0.012	Down	0.293	4.262
Blasticidin S	0.0001	Up	1.839	3.827
D-Gal alpha 1->6D-Gal alpha 1->6D-Glucose	0.030	Down	0.362	0.633
Ephedroxane	0.030	Down	0.237	4.262
Esmolol	0.016	Up	2.027	3.691
Finaconitine	0.008	Up	2.084	4.055
GW 409544	0.003	Up	1.957	4.739
alpha-Lipomycin	0.039	Up	1.828	4.041
Methyl selenac	0.006	Up	1.960	4.668
Myristoleic acid	0.005	Up	2.324	8.555
Podocarpic acid	0.015	Down	0.331	3.563
Pseudoaconitine	0.011	Up	2.184	4.725
Pyrazosulfuron	0.008	Up	2.202	4.860
trans-Crocetin (beta-D-glucosyl) (beta-D-gentibiosyl) ester	0.0002	Up	2.410	4.668

**Table 2:** Top 20 differential serum metabolites between Ang II-induced hypertension mice and control mice in ESI- mode.

**Compound**	***P*-value**	**Regulation**	**Fold change**	**Retention time/min**
(4S)-4,6-Dihydroxy-2,5-dioxohexanoate	0.050	Down	0.427	0.633
12-OPDA	0.0002	Up	2.077	8.090
13,16,19-Docosatrienoic acid	0.036	Down	0.378	9.841
4-Hydroxyphenylpyruvate	0.022	Down	0.488	3.414
4-Sulfobenzyl alcohol	0.009	Up	2.436	4.497
Callystatin A	0.021	Up	1.963	8.047
cyclo-Dopa 5-O-glucoside	0.036	Down	0.484	0.633
Deoxypeganine	0.0001	Up	5.391	6.108
Gibberellin A53 aldehyde	0.019	Down	0.397	7.156
Hinokitiol glucoside	0.006	Up	3.668	5.865
Hydrocortisone caproate	0.037	Up	2.686	7.477
Methyldopa anhydrous	0.034	Down	0.524	7.202
N(omega)-Nitro-L-arginine	0.00003	Up	42.991	0.633
Neurokinin A	0.048	Down	0.480	8.603
Pentobarbital sodium	0.025	Up	2.242	4.682
Plakinamine A	0.018	Up	3.526	7.583
Radium-224	0.030	Down	0.414	4.041
Sudan II	0.009	Up	2.287	8.339
Taxa-4(20),11(12)-dien-5alpha-acetoxy-10beta-ol	0.0008	Up	1.794	8.746
Voriconazole	0.009	Down	0.306	4.262

### Metabolic Pathway Disorders

**Figure 4:**
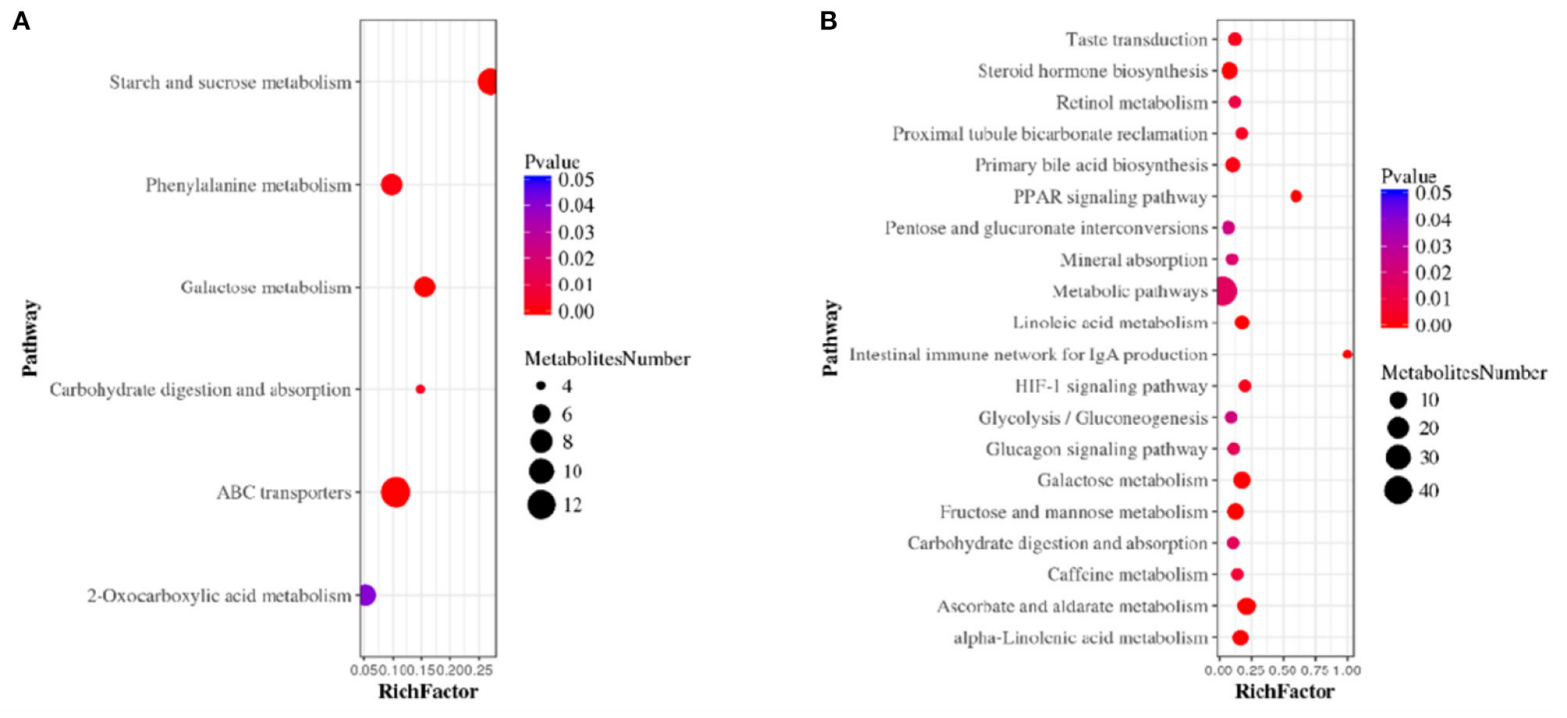
Metabolic pathway enrichment analysis in ESI+ mode **(A)** and ESI– mode **(B)**. The size of each circle represents the number of metabolites enriched in the pathway.

The serum metabolites of the mice made hypertensive by Ang II were significantly different from the control group. Compared with control mice, 581 differential metabolites were obtained under ESI+ mode and 530 under ESI– mode (Supplementary Materials). Search of a mass-based metabolomics database showed that metabolite ions detected under the ESI+ and ESI– modes included disaccharides, glycerophospholipids, amino-acids, sphingolipids, fatty acyl groups, acylcarnitines, and other organic compounds. Here, the top 20 metabolites and their regulatory changes under the two ion modes were identified as potential biomarkers in developing hypertension (Tables 1, 2). In addition, these metabolites mapped to >20 metabolic pathways, mainly sugar and linoleic acid metabolism, carbohydrate digestion and absorption, ATP binding cassette (ABC) membrane transporter transport protein, peroxisome proliferator-activated receptor (PPAR), and hypoxia-inducible factor-1 signaling pathways (Figure 4).

## Discussion

The renin-angiotensin-aldosterone system (RAAS) is one of the systems closely related to vasomotor and sodium-water metabolism *in vivo*, which plays crucial roles in cardiovascular physiology and pathophysiology (19, 20). In the RAAS, the angiotensin converting enzyme (ACE) generates Ang II, which has high bioactivity and powerful vasoconstrictor effect. It is well-known that a variety of cardiovascular risk factors are closely associated with Ang II (21–23). The ability of Ang II to elevate blood pressure is 10~40 times powerful than adrenaline (24). Ang II raises blood pressure through multiple factors, mainly through stimulating zona glomerulosa of the adrenal gland, promoting aldosterone secretion and sodium-water retention (20, 25–27). Besides, it enhances noradrenaline release from sympathetic nerve endings (28). Meanwhile, elevation of Ang II content promotes oxidative stress and endothelial dysfunction, and plays a crucial role in atherosclerosis (29, 30). Vascular damage, regulated with Ang II, is also dependent on the gut microbiome (31). It is noticed that a significant positive correlation between Ang II level and left ventricular hypertrophy in the hypertensive patients diagnosed without any treatment (32, 33). Plasma Ang II level is significantly correlated to the end diastolic interventricular septal diameter (IVSDd) in essential hypertension (34). Taken together, due to the multifactorial roles played by Ang II in the course of hypertension, it has been used in many studies to construction of hypertensive models in mice (35, 36).

In this study, we analyzed the serum metabolites of Ang II-induced hypertensive mice based on an LC-MS/MS platform. Our results showed that the slow release of Ang II induces metabolomics changes in mice, promoting the development of hypertension. According to previous research reports on Ang II-induced hypertension (37), the body weight of mice will decrease significantly during the sustained release of Ang II, which may be related to metabolic disorders in the body.

Ang II is a critical factor in hypertension, diabetes, and aging, and it induces many metabolic pathway disorders. Hypertension and diabetes are considered to be the main components of metabolic syndrome, sharing a common pathogenesis according to a large number of basic and clinical studies (38–40). About 60–70% of diabetic patients have hypertension (41), and hypertensive patients have abnormal glucose metabolism. Consistent with this, our results showed that Ang II-induced hypertension in mice was accompanied by evident glucose metabolism disorder. A significant increase in 6-phosphate-d-gluconate and decreases in maltose, lactose, and other disaccharides was found in the Ang II group. The analysis of metabolite pathway enrichment showed a disturbed glucagon signal pathway, consistent with the phenomenon of hyperinsulinemia in hypertensive patients.

In recent years, PPARs have been closely associated with energy metabolism, cell differentiation, proliferation, apoptosis, and the inflammatory response (42–44). Ang II increases the permeability of cerebral vascular endothelium *via* type 1 receptors, disrupts the membrane distribution of zonula occludens-1 and vascular endothelial-cadherin on cerebral vascular endothelium, decreases the total levels of junctional adhesion molecule-A and major facilitator superfamily domain-containing protein 2a, and increases caveolin 1 accompanied by the de-phosphorylation of PPARα. PPARα agonists improve the endothelial permeability caused by Ang II (45). Interestingly, in ESI– mode, we found that the levels of three metabolites [9-cis-retinoic acid, 9(S)-hydroxyoctadecadienoic acid (HODE), and 13(S)-HODE] are closely associated with the PPAR signaling pathway. After 2 weeks of Ang II induction in mice, the content of 9-cis-retinoic acid, an active metabolite of vitamin A, increases significantly. The content of 9-cis-retinoic acid in the serum of Ang II-induced hypertensive mice was 1.42 times compared to control mice (*P* = 0.03; VIP = 1.66). 9-cis-Retinoic acid is an active retinoid that regulates expression of retinoid responsive genes (46). 9-cis-Retinoic mediates gene transcription acting through the retinoic acid receptors (RARs) and the retinoid X receptors (RXRs) in cells (47). In addition to PPARs, RXRs are also an essential heterodimeric partner for other subclass I nuclear receptors, such as the farnesoid X receptor (FXR), thyroid hormone receptors, the vitamin D receptor and the liver X receptor (LXR) (48–51). However, the transcriptional complex formed by RXRs and PPARs plays a critical role in energy balance, such as glucose homeostasis, fatty acid handling and triglyceride metabolism (52). The PPAR-RXR transcriptional complex also participates in inflammatory and vascular responses in endothelial and vascular smooth muscle cells directly (52–54). It has been suggested that RXR regulates the growth and differentiation of normal and malignant cells, and inhibits the prostaglandin expression of endoperoxide-2 (55). However, retinoic acid is a toxin that can bring about fracture, skin injury and swelling, serum calcium elevation, limited dose hyperlipidemia (cholesterol and triglyceride elevation), and hypothyroidism (56). Ang II contributes to an increase in the content of two HODEs that have been used as biomarkers for assessing oxidative status (57).

The ABC is an outflow promoter of phospholipid and cholesterol, playing an essential role in the development of atherosclerosis and arterial hypertension. ABCA1 mediates the first step of reverse cholesterol transport by transporting excess cholesterol in peripheral tissues to the liver for excretion (58). Recent evidence has shown that the expression of ABCA1 is significantly decreased in patients with hypertension, and the outflow of cholesterol to apo-A1 leads to increased carotid intima-media thickness, and promotes arterial hypertension (59). Interestingly, in ESI+/ESI– modes, we identified 19 differential metabolites that were enriched in the metabolic pathway of the ABC transporter. These substances may affect lipid metabolism and increase blood vessel wall pressure by interfering with the flow of cholesterol from monocytes, macrophages, and the liver.

In this study, we found changes in the serum metabolome treated with Ang II in mice, providing new clues for the further study of the pathophysiological mechanisms in hypertension. Our non-targeted metabolomics research identified specific differences related to carbohydrate, lipid, and carbohydrate metabolism in hypertension. These results improve the understanding of systemic metabolic response to sustained release of Ang II in hypertensive mice, providing a new panel of biomarkers that may be used to predict blood pressure fluctuations in the early stages of hypertension, although researches about the clinical use of these metabolites as potential biomarkers in hypertension is still needed.

## Data Availability Statement

All relevant data are within the paper and its Supplementary Materials. The metabolomic data for this study can be found in the MetaboLights database (60) under Accession No. MTBLS2643 (http://www.ebi.ac.uk/metabolights/MTBLS2643). The full dataset is also available from Tingting Zhou (tingtingchou@126.com).

## Ethics Statement

The animal study was reviewed and approved by the Animal Experimentation Ethics Committee, Jiangnan University (License No: JN. No 20190930c1000120[232]).

## Author Contributions

SY and ZW: conception, design, data analysis, and interpretation. MG, MD, XW, and FY: administrative support. LG, LL, and YL: provision of study materials or patients. LF and TZ: collection and assembly of data. All authors manuscript writing, final approval of manuscript, read, and approved this manuscript.

## Conflict of Interest

The authors declare that the research was conducted in the absence of any commercial or financial relationships that could be construed as a potential conflict of interest.

## Abbreviations

RAAS: renin-angiotensin-aldosterone system
Ang II: angiotensin II
UPLC-Q-TOF/MS: ultra-performance liquid chromatography-quadrupole time-of-flight mass spectrometry
LC-MS: liquid chromatography-mass spectrometry
QC: quality control
ESI+: electrospray ionization positive ion mode
ESI–: electrospray ionization negative ion mode
RSD: relative standard deviation
PCA: principal component analysis
PLS-DA: partial least squares-discriminant analysis
ABC: ATP binding cassette membrane transporter
PPAR: peroxisome proliferators-activated receptor.
